# RETRACTED: Cegolon et al. Primary SARS-CoV-2 Infections, Re-Infections and Vaccine Effectiveness During the Omicron Transmission Period in Healthcare Workers of Trieste and Gorizia (Northeast Italy), 1 December 2021–31 May 2022. *Viruses* 2022, *14*, 2688

**DOI:** 10.3390/v18111258

**Published:** 2026-11-12

**Authors:** Luca Cegolon, Corrado Negro, Giuseppe Mastrangelo, Francesca Larese Filon

**Affiliations:** 1 Department of Medical, Surgical & Health Sciences, University of Trieste, 34137 Trieste, Italy; 2 Occupational Medicine Unit, University Health Agency Giuliano-Isontina (ASUGI), 35128 Trieste, Italy; 3 Senior Researcher, University of Padua, 35128 Padua, Italy

The journal retracts the article titled “Primary SARS-CoV-2 Infections, Re-infections and Vaccine Effectiveness during the Omicron Transmission Period in Healthcare Workers of Trieste and Gorizia (Northeast Italy), 1 December 2021–31 May 2022” [1], cited above.

Following publication, the authors notified the Editorial Office of a significant error in the calculation of incidence rates used in the study [1].

In accordance with the journal’s standard procedures, the Editorial Office and Editorial Board conducted an investigation, which determined that the reported error significantly affected the statistical analysis and the validity of the article’s findings. While the authors cooperated with the investigation and proposed revisions, the Editorial Board concluded that the extent of the necessary revisions exceeded what could be adequately addressed through a standard correction. Consequently, the Editorial Board has decided to retract this article [1] in accordance with MDPI’s retraction policy.

This retraction was approved by the Editor-in-Chief of *Viruses*.

The authors have been informed of this retraction and have indicated their agreement with this decision.
